# *TCF7L2 *and therapeutic response to sulfonylureas in patients with type 2 diabetes

**DOI:** 10.1186/1471-2350-12-30

**Published:** 2011-02-24

**Authors:** Andreas Holstein, Michael Hahn, Antje Körner, Michael Stumvoll, Peter Kovacs

**Affiliations:** 1 First Department of Medicine, Clinic Lippe-Detmold, Detmold, Germany; 2 University Hospital for Children and Adolescents, University of Leipzig, Leipzig, Germany; 3 Department of Medicine, University of Leipzig, Leipzig, Germany; 4 Interdisciplinary Centre for Clinical Research, University of Leipzig, Leipzig, Germany

## Abstract

**Background:**

Variants in the *TCF7L2 *have been shown to be associated with an increased risk for type 2 diabetes (T2D). Since the association with diabetes could be explained by effects on insulin secretion, we investigated whether patients with diabetes risk alleles at rs7903146 might have an altered hypoglycaemic response to sulfonylureas (SUs).

**Methods:**

We recruited 189 patients with T2D being treated with SUs and determined the rs7903146 diabetes risk genotype. We used a logistic regression with secondary SU failure defined as an A1C ≥7.0% after 6 months of SU treatment.

**Results:**

In univariate regression analyses, *TCF7L2 *genotype was the only predictor of SU treatment failure. The rs7903146 T allele was significantly more frequent in the group of patients who failed to respond to SU (36%) than in the control group (26%) [*P *= *0.046; *odds ratio (OR): 1.57 (1.01-2.45) in an additive mode of inheritance].

**Conclusions:**

Our data suggest that patients with diabetes risk alleles in *TCF7L2 *have an altered hypoglycaemic response to SUs resulting in earlier secondary failure.

## Background

The TCF7L2-gene (*TCF7L2*; Transcription factor 7-like 2) encodes a transcription factor (Tcf-4) that is involved in the regulation of cellular proliferation and differentiation [1]. Variants in the *TCF7L2 *have initially been shown to be associated with an increased risk for type 2 diabetes (T2D) in a genome-wide analysis of the isolate population of Iceland [2]. The strongest associations with T2D with a clear gene dose effect were reported for the rs7903146 variant [3]. The initial findings have been replicated in independent studies in multiple ethnic populations and were summarized in a large global meta-analysis [4]. The risk alleles actually predicted the progression from impaired glucose tolerance to diabetes prospectively [5] and an increased severity of the disease [6] in adults. Also, *TCF7L2 *variants conferred a higher risk for early impairment of glucose metabolism emerging as soon as in childhood and adolescence [7]. Some clinical data suggested that the polymorphisms affected the capacity of pancreatic β-cells to secrete insulin rather than aggravating insulin resistance [5,8-13], possibly by impaired β-cell proinsulin-processing [14]. This was further supported by expression data suggesting a putative role of *TCF7L2 *in β-cell differentiation [12]. Considering the role of *TCF7L2 *risk variants in insulin secretion, Pearson et al. [15] hypothesized that patients with diabetes risk alleles at rs12255372 and rs7903146 have an altered hypoglycaemic response to sulfonylureas (SUs) due to decreased β-cell function. They could indeed show that carriers of the diabetes risk alleles from the GoDART (Genetics of Diabetes Audit and Research Tayside) study were less likely to respond to SUs [15]. This study suggested that genetic variation in *TCF7L2 *can alter response to therapy in T2D. Since a causal phenotype-genotype relationship can not be established with one initial report, replication studies are the backbone to the genetic epidemiology of complex diseases [16], and play a crucial role in pharmacogenomics as well.

Therefore, we sought to evaluate the association between genetic variants in *TCF7L2 *with SU treatment failure in an independent cohort from Germany. We recruited 189 patients with T2D being treated with SU agents and determined the *TCF7L2 *rs7903146 diabetes risk genotype, which has been reported as having the strongest association with T2D [3]. We used a logistic regression with secondary SU failure defined as an A1C ≥7.0% after 6 months of SU treatment.

## Methods

### Subjects

One hundred and eighty-nine patients with T2D, all of them being treated with SU agents, were recruited at the medical department of the Klinikum Lippe-Detmold, a large tertiary care hospital in East Westphalia, Germany, between 1 January 2000 and 30.11.2009. As the only hospital in the area, the one at Lippe-Detmold is responsible for the inpatient and outpatient management of all emergencies in the region. All patients had been treated with the SU drugs glimepiride (n = 147), glibenclamide (n = 39) and gliquidon (n = 3). Ninety-seven patients failed to respond to SU treatment according to our definition of A1C ≥7.0% after 6 months of treatment (76 patients treated with glimepiride, 19 with glibenclamide and 2 with gliquidon). Forty six patients were additionally treated with insulin. The mean (± SD) daily dose of SU agents was comparable between subjects who failed to respond to SUs and the controls (5.0 ± 3.7 mg vs. 6.8 ± 3.7 mg, *P *= 0.13 for glibenclamide; 2.5 ± 1.6 mg vs. 2.5 ± 1.4 mg for glimepiride, *P *= 0.99). As our patients were recruited within the framework of a study originally investigating the risk of hypoglycaemia [17], eighty nine patients had experienced a severe hypoglycaemia, which was defined as a symptomatic event requiring treatment with intravenous glucose and was confirmed by a blood glucose measurement of <50 mg/dl (<2.8 mmol/l). Seventy-two subjects were additionally treated with insulin sensitizing drug metformin (32 patients in the control group and 40 patients in the group of patients with SU treatment failure; *P *= 0.36, Table 1). The protocol was approved by the Ethics Committee of the University of Münster School of Medicine and by the Ethics Committee of the University of Leipzig, School of Medicine. All patients gave written informed consent to participate in the study.

**Table 1:** Clinical characteristics of all participants.

	Treatment with sulfonylurea (A1C <7%)	Failure of treatment with sulfonylurea (A1C≥7%)	*P-value*
	N = 92	N = 97	
**Gender **(Male/Female)	45/47	47/50	0.95*
**Age **(yr)	78.2 ± 9.6	78.3 ± 9.0	*0.98*
**BMI **(kg/m^2^)	26.8 ± 5.2	27.0 ± 4.7	*0.71*
**Creatinine **(mg/dl)	1.73 ± 1.20	1.52 ± 0.55	*0.13*
**Creatinine clearence **(ml/min)	42.96 ± 22.61	42.10 ± 18.77	*0.79*
**A1C **(%)	6.13 ± 0.51	7.65 ± 1.40	*<0.001*
**Age at onset of diabetes **(yr)	68.0 ± 13.4	66.0 ± 10.9	*0.28*
**Diabetes duration **(yr)	10.2 ± 9.5	11.9 ± 8.4	*0.20*
**Co-medication **(n all drugs)	7 ± 3	7 ± 3	*0.29*
**Sulfonylurea daily dose **(mg)	3.68 ± 6.40	4.24 ± 6.85	*0.56*
**Metformin treatment **(n patients)	32	40	0.36*

Data are mean ± SD; *P*- values for comparisons between genotypic groups by ANOVA statistics;

*- χ^2 ^test.

### Genotyping of rs7903146

Genotyping of rs7903146 in all study subjects was done using the TaqMan allelic discrimination assay (Assays-on-Demand (TM), SNP Genotyping Products; Applied Biosystems, Inc.) on an ABI PRISM 7500 sequence detector (Applied Biosystems Inc.) according to the manufacturer's protocol. The genotype distribution was consistent with Hardy-Weinberg equilibrium (*P *> 0.05). Genotyping success rate was >99%, and duplicate genotyping concordance was 100%.

### Statistics

Standard descriptive and comparative statistics (χ^2 ^test, t-test) were used to characterize and compare clinical parameters in different groups (controls, cases). Logistic regression analyses were used to calculate the effects of investigated factors on SU treatment failure, which were reported as odds ratio with 95% CI (confidence intervals). In the additive model, homozygotes for the major allele, heterozygotes and homozygotes for the minor allele were coded to a continuous numeric variable for genotype (as 0, 1, 2). Data were analyzed using the SPSS software package (version 15.0; SPSS, Inc., Chicago, IL).

## Results

Clinical characteristics of all study participants are given in Table 1. As expected, the subjects with failure of SU treatment had a higher A1C than the controls (Table 1). However, both groups were comparable in regard to the age, gender, age at onset of diabetes, duration of diabetes and creatinine clearance and co-medication presented as number of drugs taken by the patient (Table 1). Also the number of patients additionally treated with metformin was similar between the groups (Table 1).

In the univariate logistic regression analyses, the *TCF7L2 *genotype was the only predictor of SU treatment failure (Table 2). The rs7903146 T allele was significantly more frequent in the group of patients who failed to respond to SU (36%) than in the control group (26%) [*P = 0.046; *odds ratio (OR): 1.57 (1.01-2.45) in an additive mode of inheritance] (Table 3). In the control group, 56.0% of subjects had the CC, 36.3% had the CT and 7.7% had the TT genotype. Among patients who failed to respond to SUs, 41.2% were CC homozygous, 46.4% were CT heterozygous and 12.4% were TT homozygous (Table 3).

**Table 2:** Univariate regression analyses of predictors on failure of sulfonylurea treatment in patients with type 2 diabetes.

	**Odds ratio **(95% CI)	*P-value*
**Gender**	0.98 (0.56-1.74)	*0.95*
**Age **(yr)	1.00 (0.97-1.03)	*0.98*
**Diabetes duration **(<5 yrs vs. >5 yrs)	1.65 (0.86-3.17)	*0.13*
**Sulfonylurea daily dose **(mg)	1.01 (0.97-1.06)	*0.57*
***TCF7L2 *genotype **(rs7903146; per allele effect)	1.57 (1.01-2.45)	*0.046*
***TCF7L2 *genotype **(CC vs. TT)	2.09 (1.02-4.27)	*0.043*

**Table 3:** Effect of rs7903146 genotype on sulfonylurea treatment failure under logistic regression analysis.

Genotype rs7903146	Total	Controls (A1C <7%)	Failure of treatment with sulfonylurea (A1C≥7%)	Frequency of T-allele (controls vs. treatment failure)	Additive *P* value OR (95% CI)
	n = 188	n = 91	n = 97		

CC	91 (48.4%)	51 (56.0%)	40 (41.2%)		
CT	78 (41.5%)	33 (36.3%)	45 (46.4%)	0.26/0.36	***0.046*** 1.57 (1.01-2.45)
TT	19 (10.1%)	7 (7.7%)	12 (12.4%)		

To investigate whether the rs7903146 effect is specific to the mechanism of action of SUs, we evaluated the genotype effects on response to a non-insulin secretagogue metformin. By analysing 72 metformin-treated individuals only, no effect of the genotype on SU treatment failure was found [*P *= 0.98; OR 1.01 (0.50-2.03)].

### Secondary confirmatory analyses

In secondary analyses we used a logistic regression with secondary SU failure defined as the addition of insulin after at least 6 months of SU therapy and corresponding A1C measurement of ≥7.0%. Based on these criteria 46 patients from our cohort failed to respond to SU treatment and were additionally treated with insulin.

In the univariate logistic regression analyses including 46 patients who failed to respond to SU treatment and 143 control subjects, diabetes duration (<5 yrs vs. >5 yrs) appeared to be the strongest predictor of SU treatment failure [OR: 4.06 (1.50-11.01), *P *= 0.006]. We also assessed the effect of rs7903146 on SU treatment failure. The rs7903146 T allele was significantly more frequent in the group of patients additionally treated with insulin (40%) than in the control group treated only with SUs (28%) [*P *= 0.03; odds ratio (OR): 1.73 (1.06-2.84) in an additive mode of inheritance]. In the control group, 53% of subjects had the CC genotype, 39% had CT and 8% had TT. Among patients treated with insulin, 35% were CC homozygous, 50% were CT heterozygous and 15% had the TT genotype. The results remained materially unchanged even after including diabetes duration as a strong predictor of SU treatment failure in these analyses, thus indicating an independent effect of the genotype [OR: 1.66 (0.99-2.79), *P *= 0.06 in additive model].

## Discussion

In the present study, we investigated the effect of *TCF7L2 *diabetes risk T-allele at rs7903146 on therapeutic response to SUs. In univariate regression analyses, *TCF7L2 *genotype was the only predictor of SU treatment failure. The rs7903146 T-allele conferred a higher risk for sulfonylurea treatment failure as it was significantly more frequent in the group of patients who failed to respond to SUs (36%) than in the control group (26%). After adjusting for diabetes duration the odds ratio did not change (OR = 1.57) and the *P*-value reduced just minimally (from *P *= 0.046 to *P *= 0.057), thus indicating independent effect of the *TCF7L2 *genotype. Despite the smaller sample size and so, limited statistical power, our data are in line with findings reported by Pearson et al. [15], suggesting that variation in *TCF7L2 *influences therapeutic response to SUs. Pearson et al. observed that homozygous carriers of the *TCF7L2 *risk alleles (rs1225372 and rs7903146) were twice as likely not to respond to SUs as patients homozygous for the non-risk alleles [15]. Considering pretreatment A1C levels as covariate in logistic regression analyses even strengthened the association between sulfonylurea response and genotype at rs7903146 [15]. Even though the findings are consistent between the present study and that reported by Pearson et al., several differences should be noted. First, Pearson et al. investigated a total of 911 SU users of 4,469 patients with T2D from the DARTS/MEMO (Diabetes Audit and Research Tayside/Medicines Monitoring Unit) collaboration database, who were recruited to GoDARTS between 1997 and 2006 [15]. Second, SU failure was defined very restrictively as an A1C >7% within 3-12 months after treatment initiation. According to these criteria, 42% of SU users failed to respond to the therapy [15]. In our study we chose a comparable, overlapping definition of secondary SU failure with at least 6 months of SU therapy and corresponding A1C measurement of ≥7.0%. According to this definition, 51% of our patients failed to respond to SU therapy. We are aware that the difference in SU treatment failure frequency may be due to various study designs but it is noteworthy that to date, there is no widely accepted definition of secondary SU failure. Due to the lack of uniform definition, the frequency of SU failure varies considerably between 22% and 50% after 12 and 36 months of treatment, respectively [18,19]. The decreasing effectiveness of SUs results from progressive loss of β-cell function but also from patient related factors (dietary incompliance, weight gain, lack of exercise). Despite the above mentioned differences between the studies, frequencies of TT homozygous subjects in the groups of patients who failed to respond to the therapy (independent of definition) were significantly higher than in the control groups and were comparable between both studies (12% vs. 8% in the German patients and 16% vs. 8% in the GoDARTS study). Also, genotype distribution of the rs7903146 was similar, with 10% and 11% of diabetic population with 2 copies of the T-allele in our study and the GoDARTS study, respectively. Finally, similarly to the GoDARTS study, our data suggest that carriers of the T allele were 57% more likely to fail SU treatment; TT homozygotes were twice as likely as CC homozygotes.

It is noteworthy that an alternate definition of SU treatment failure in our cohort based on addition of insulin after at least 6 months of SU therapy and corresponding A1C measurement of ≥7.0% yielded similar results. Even though not independent from the previous analyses, these findings provide further support for the role of *TCF7L2 *genotypes in altered hypoglycaemic response to SUs. Interestingly, when using this definition of SU treatment failure, diabetes duration appeared to be a predictor of treatment failure along with the *TCF7L2 *genotype. Nevertheless, the genotype effect was independent as even after adjustment for diabetes duration, the results remained materially unchanged. Although the *P*-value went from 0.04 to 0.06 the odds ratio reduced only minimally (from 1.73 to 1.66).

Indirectly, our findings also support studies that favour the role of *TCF7L2 *in the regulation of insulin secretion. However, we are aware that since the *TCF7L2 *variants increase progression from IGT to diabetes [5], additional models considering diabetes therapy, particularly including a control group having been treated with a different antidiabetic agent - e.g., metformin, would be desirable to clarify whether the observed data reflect pharmacogenetic effects specific to SUs or rather a disease-genetic process. Indeed, such a control group treated with metformin was included in the GoDARTS study [15]. The study suggested pharmacogenetic effects of *TCF7L2 *SNPs influencing therapeutic response to sulfonylureas but not metformin, since no association was seen between metformin response and *TCF7L2 *variants [15]. Even though limited by the small sample size (N = 72), we also failed to observe any influence of *TCF7L2 *genotypes on the response to metformin, as a non-insulin secretagogue, thus further supporting the notion that *TCF7L2 *effect is specific to the mechanism of action of SUs.

One of the major limitations of our study is the low sample size and so, limited statistical power. Taking into account genotype frequencies and the sample size in our study we had a statistical power of 80% (at α = 0.05) to detect genetic risk (odds ratio) of 1.8 for treatment failure in additive mode of inheritance (software Quanto version 1.2.2) [20]. In contrast, the GoDARTS study by Pearson et *al*. [15] had 80% power to detect risk (OR) as low as 1.3. Also noteworthy, given the *TCF7L2 *diabetes risk genotypes make patients more resistant to the action of sulfonylureas, one would expect that carriers of the risk genotype should be less likely to become hypoglycaemic. However, in our study we could not observe any differences in the frequency of the diabetes risk allele between patients with and without hypoglycaemia (*P *= 0.30).

## Conclusion

In conclusion, present data strengthen previously reported findings suggesting altered therapeutic response to SUs in patients with T2D carrying the diabetes risk alleles at *TCF7L2 *variants.

## Competing interests

The authors declare that they have no competing interests.

## Authors' contributions

MH was responsible for clinical characteristics of study subjects. AK and MS edited the manuscript and contributed to the discussion. AH and PK conceived and designed the study and wrote the manuscript. All authors read and approved the final manuscript.

## Pre-publication history

The pre-publication history for this paper can be accessed here:

http://www.biomedcentral.com/1471-2350/12/30/prepub
